# Analyzing the potential of waste cooking oils as biolubricants for electric vehicles†

**DOI:** 10.1039/d5ra08832a

**Published:** 2026-03-09

**Authors:** Seshasai N. Ayyadevara, Pial Das, Majher I. Sarker, Brajendra K. Sharma, Sougata Roy

**Affiliations:** a Department of Mechanical Engineering, Iowa State University Ames IA USA sroy@iastate.edu; b Sustainable Biofuels and Coproducts Research Unit, USDA-ARS-NEA-ERRC Wyndmoor PA USA majher.sarker@usda.gov

## Abstract

The transition from internal combustion engines (ICE) to electric vehicles (EVs) has pushed the search for new sustainable lubricants that can withstand relevant lubrication challenges, like high torque loads and stray currents in drivetrains, which can accelerate oxidation and increase component wear, posing a critical challenge for electric powertrain components. Conventional Automatic Transmission Fluids like ATF III and ATF V have shown different wear mechanisms and coefficient of friction (CoF) trends in the presence of simulated stray currents. While many studies have focused on vegetable oils as biobased oils, waste cooking oils (WCO) offer a more sustainable alternative, yet their performance under electrified conditions is yet to be explored. In this study, four WCO samples were collected from different sources and evaluated against regular soybean oil (RSO) through structural, physico–chemical, and tribological analysis under electrified and unelectrified sliding conditions. Structural analyses using FTIR, ^1^H/^13^C NMR, GC-MS, and CMS confirmed the triglyceride integrity across all samples, with differences in fatty acid composition influencing physicochemical properties. WCO B-4, with the lowest unsaturation of 3.43 C=C bonds/triglyceride, exhibited the highest viscosity. At the same time, WCO B-2 showed higher oxidation resistance due to high oleic acid content (66.2%) and a lower degree of unsaturation of 3.73 compared to other WCOs, which reduced reactive oxidation sites. WCO B-2 exhibited the lowest cloud and pour points, which can be attributed to the presence of low saturated fatty acids in triglyceride molecules, and is dominated by monounsaturated fatty acids. Tribological testing on aluminum–steel contacts showed that, under unelectrified conditions, WCO B-3 and B-4 resulted in reduced average coefficients of friction by 18% and 23%, respectively, and had lower average wear depth compared to RSO. Under electrified conditions, all batches of lubricants exhibited increased wear and oxidation, yet WCO B-4 maintained the lowest wear depth despite frictional instability. Additional surface characterization *via* high-resolution microscopy and spectroscopy techniques confirmed more severe oxidation and lower material transfer under current, underscoring the degradation risk in electrically stressed contacts.

## Abstract 2

Evaluating the tribological behavior of filtered waste cooking oils against that of regular soybean oil under electrified and unelectrified conditions to assess the influence of electrification on surface protection under lubricated conditions.

## Graphical abstract

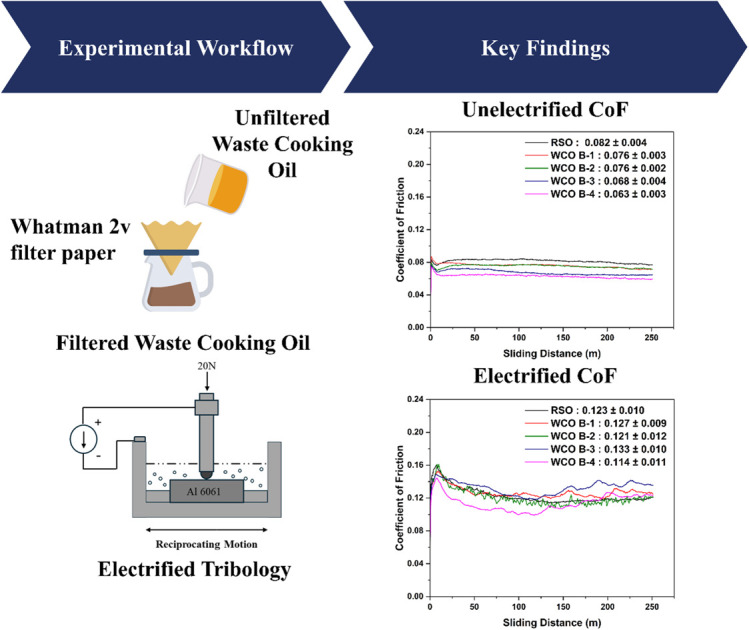

## 1. Introduction

An average passenger vehicle emits about 4.6 metric tons of CO_2_ annually, along with trace amounts of methane (CH_4_) and nitrous oxide (N_2_O).^1^ Vehicles powered by gasoline and diesel significantly affect air quality and contribute to environmental degradation.^2^ In response to increasing environmental concerns, automobile manufacturers are transitioning from ICEs to electric vehicles (EVs) as a clean alternative to reduce fossil fuel dependency, and environmental regulations have also become stricter, with limited fossil fuel reserves.^3,4^ The first concept of an electric motor that is capable of turning machinery dates back to the 1830s, and commercial EV sales started in the late 19th century.^5^ Modern EVs come in several types: battery electric vehicles (BEVs) solely operate on electricity stored in batteries and can be charged through the power grid; hybrid electric vehicles (HEVs) combine an internal combustion engine with a battery powered motor, and these vehicles cannot be plugged in for charging, they instead recharge the battery through regenerative breaking; plug-in hybrid electric vehicles (PHEVs) include both a battery and an internal combustion engine. Unlike HEVs, they can be charged using an external power source, and fuel cell electric vehicles (FCEVs) use a hydrogen cell to generate electricity inside the vehicle. The only emission from FCEVs is water vapor and warm air.^6,7^

In the first quarter of 2025, about 22% light-duty vehicles sold in the United States were EVs.^8^ EVs use electric motors powered by batteries or fuel cells, converting electrical energy to mechanical energy with zero tailpipe emissions while running on electricity.^9^ Compared to ICE vehicles, only 21.5% of the total fuel energy supplied to an ICEV is used to move the vehicle, whereas 77% of the total energy from the electric motor is used to move an EV, which makes it 3.6 times more efficient.^10,11^ Due to the higher energy conversion efficiency of electric motors and their associated power electronics compared to internal combustion engines, EVs require less energy to operate.^12^ Although EVs are generally more energy efficient and economically friendly than ICE vehicles, electric powertrains are subjected to higher torque loads,^13^ increased rotational speeds, stray currents, and voltages. These currents have a negative effect on the tribological performance of the mechanical parts, like bearings and gears in the powertrain.^14^ Conventional Automatic Transmission Fluids (ATFs) used in EV powertrains pose sustainability concerns as they are non-biodegradable and contain harmful chemical additives.^15^

From electric vehicles to biomedical implants, lubricants play a critical role in controlling the efficiency of the systems. 23% of the world's total energy consumption (119 EJ) is from tribological contacts, where 20% is utilized to overcome friction (103 EJ) and 3% is used to remanufacture worn parts due to wear and wear-related failures (16 EJ).^16^ The domestic consumption of motor and industrial lubricants was 2.47 billion gallons in the United States, from which 1.37 billion gallons of used oil could be recycled.^17^ Improper disposal or accidental spills of these fluids can result in significant environmental harm.^18^ Used oil from a single oil change could contaminate one million gallons of fresh water.^19^ With the transition from ICE vehicles to EVs and with the harsh operating conditions, the demand for more durable and reliable lubricants has grown in recent years. Previous studies have shown that ATFs like ATF Type III and ATF Type V showed increased coefficient of friction and had different wear mechanisms when exposed to electricity during tribology testing to simulate stray currents.^20^ Studies have also shown that contact surfaces suffer severe oxidation and oxidation wear under electrification.^21^ Copper corrosion resistance, thermal conductivity, electrical conductivity, and viscosity of the lubricant play a major role in the electric drive train, as the lubricant in contact with motors should not corrode windings. A fluid with high thermal conductivity would keep the motor cool. On the other hand, if the electrical conductivity of the lubricant is too low, charge can accumulate and discharge as electrostatic discharge, damaging bearings and other parts. If the discharge is too high, leakage currents will be increased, causing insulation stress/efficiency losses.^22–26^ These findings prompted researchers to investigate environmentally friendly alternatives and find their potential in maintaining performance under electrified conditions.

Recycling waste cooking oil (WCO) for the production of biodiesel in the presence of different chemical modifications has been explored in previous research.^27,28^ This opens an opportunity to explore the characteristics of WCOs as biobased lubricating oil, especially for electric vehicles, since the tribological properties of WCOs remain unexplored in both unelectrified and electrified conditions. Numerous studies have been pursued to explore the tribological characteristics of various natural oils, such as coconut oil,^29^ rapeseed oil,^30^ castor oil^31^ jatropha oil,^32^ canola oil,^33^ sunflower oil^34^ and palm oil.^35^ Previous investigations have presented how chemically modified soybean and high oleic soybean oil with additives improved their performance as lubricants.^36–38^ Common oils used in cooking, according to the USDA, are canola oil, corn oil, cottonseed oil, grapeseed oil, olive oil, peanut oil, safflower oil, soybean oil, sunflower oil, sesame oil, and walnut oil.^39^ Since the oils are from different sources, it would give us an idea of how different blends of used oil would change the tribological properties. Hence, regular soybean oil was used as a reference biobased lubricant to compare the tribological behavior of refined waste cooking oils captured from various sources. This investigation will build the framework for future investigations focused on chemical modifications of these WCOs and their formulations with novel lubricant additives for potential applications in future EVs.

## 2. Materials and methods

### 2.1. Materials

All four waste cooking oil (WCO) samples were obtained from four different local restaurants, followed by processing them *via* gravitational filtration with Whatman 2v filter paper at an ambient temperature. The oil samples were kept in the refrigerator and utilized for analysis without any additional purification. The samples were named as Waste Cooking Oil Batch-1 (WCO B-1) through Batch-4 (WCO B-4). One commercially available refined, bleached and deodorized (RBD) soybean oil was used as a baseline reference. Aluminum 6061-T6 bars were purchased from McMaster-Carr and sectioned into blocks measuring 1 inch × 1 inch with a thickness of 0.25 inch. AISI 52100 steel balls with a diameter of 6 mm were procured from McMaster-Carr. These two different materials were selected to study tribological behavior at the sliding interface under various lubricant conditions. All lubricants (*i.e.*, regular soybean oil and different batches of waste cooking oils) were sonicated for 30 minutes before each tribological test using an ultrasonic bath.

### 2.2. Fourier transform infrared analysis

A Bruker Infrared (IR) spectrometer was utilized for the IR analysis of WCOs. The Fourier transform infrared (FTIR) setup featured a platinum diamond crystal for attenuated total reflectance infrared (ATR-IR). The spectra were obtained by performing sixty-four scans with a resolution of 4 cm^−1^ across the span of 4000–500 cm^−1^.^40^

### 2.3. Nuclear magnetic resonance (NMR)

The structural characterization of the samples was conducted with a 14 tesla NMR spectrometer (Agilent Technologies, Santa Clara, CA). This spectrometer features a 5 mm One NMR probe. Every sample was solubilized in deuterated chloroform (Cambridge Isotopes Laboratories, Andover, MA). A relaxation delay lasting 2 seconds, an acquisition duration of 2.28 seconds, and a pulse angle of 45° were employed at 25 °C to obtain the ^1^H spectrum. The spectral width for the ^1^H spectrum was 12 parts per million (ppm). The ^13^C NMR spectra resulted from an average of 1000 transients, employing a pulse angle of 45°, with a subsequent relaxation delay of 2 seconds, and an acquisition time of 0.87 seconds. The observed spectra exhibited spectral widths measuring 253 ppm. The solvent resonances served as internal references for chemical shifts. The spectra underwent processing through SpinWorks4 software (version 4.2.10), created by Kirk Marat, University of Manitoba, Canada.

### 2.4. Gas chromatography-mass spectroscopy (GC-MS)

To ascertain the composition of fatty acids in oil samples, WCO B-1, WCO B-2, WCO B-3, WCO B-4, and RSO, a Gas Chromatograph (model 8890N, Agilent Technologies) was utilized. The Gas Chromatography employed for this analysis was fitted with a mass selective (MS) detector (Agilent model number: 5977N). Furthermore, a column (Supelco SP-2380) measuring 30 m in length, with a diameter of 0.25 mm and a film thickness of 0.2 µm, was used. In the context of GC-MS analysis, the oil samples underwent transesterification to generate fatty acid methyl esters (FAMEs). Approximately 100 mg of each oil sample was placed separately in 20 mL reaction vials. Subsequently, 15 mL of 2% sulfuric acid (H_2_SO_4_) in methanol was introduced. After sealing the reaction vials, they were agitated for 2 hours at a temperature of 80 °C. The mixture of reaction was then allowed to reach room temperature (RT), after which the methanol was removed through evaporation using vacuum conditions. The resulting mixture underwent an extraction procedure utilizing ethyl acetate to isolate the target FAME. The unrefined product was subjected to washing three times using 15 × 3 mL water to remove any residual glycerol and acid catalyst from the FAME. The FAME containing ethyl acetate extract underwent further processing by being washed with 5 mL of brine solution. Following this, anhydrous sodium sulfate (Na_2_SO_4_) was used to dry the extract. A volume of 5 µL of FAME was diluted with ethyl acetate (∼1 mL) and then injected into the GC-MS machine. The column underwent initial heating to a temperature of 70 °C and was maintained at this level for a duration of 2 minutes. Thereafter, the temperature of the column was increased to 250 °C at a pace of 20 °C min^−1^. After achieving 250 °C, it was kept constant for 10 minutes. Helium served as the carrier gas, maintaining a flow rate of 1.5 mL min^−1^. A ratio of 50 : 1 was applied for the split. The temperatures of the injector and detector were set at 230 °C and 280 °C, respectively.

### 2.5. Compact mass spectroscopy (CMS)

The Advion (Ithaca, New York) Expression Compact Mass Spectrometer (CMS) was employed to ascertain the molecular weight of oils, WCO B-1, WCO B-2, WCO B-3, WCO B-4, and RSO, along with their respective fragments. In the CMS, there exists a singular quadrupole mass analyzer that is integrated with an atmospheric pressure interface, enabling both atmospheric pressure chemical ionization (APCI) and electrospray ionization (ESI) functionalities, allowing for polarity switching between negative and positive during a singular analytical run. The CMS is capable of mass measurements across a range of 0–2000 *m*/*z*. The expression CMS enables swift verification and recognition of compounds in both reverse and normal phase chromatographic settings, compatible with supercritical fluid and ultra-performance liquid chromatographic methods.

In this study, the ESI source was utilized for data collection. The temperature and voltage of the capillary were set to 300 °C and 80 volts, respectively. Nitrogen served as the source gas, maintained at 350 °C and a pressure of 4.8 × 10^−3^ mBar. Methanol (HPLC grade) was used as the mobile phase to be flown at a span of 0.5 mL min^−1^. The mass acquisition range was configured from 250–2000 *m*/*z*, with a scan time and speed of 1000 ms and 1750 *m*/*z* per sec, respectively. A sample volume of 20 µl was inserted into the APCI ion source for assessment, with a concentration of approximately 10–50 ppm.

### 2.6. Pressure differential scanning calorimetry (PDSC)

The DSC thermograms for the oil samples were obtained using a Q20 TA Instruments device (New Castle, DE, USA). During the experimental process, around 1.5–2.0 mg of the oil sample was accurately measured and placed into a pan of aluminum. To enhance the interaction between the sample and the dry air (reactant gas), the pan was closed with pinhole lids. By employing regulated gas dispersion through a pinhole, the contact between the sample and the dry air enabled the oil sample to become saturated with air while successfully inhibiting its volatilization. The thickness of the oil film in the pan must remain below 1 mm to ensure effective oil/air contact and to eliminate any discrepancies in the results caused by limitations in gas diffusion. The temperature calibration was performed using indium metal, which has a melting point of 156.6 °C, while maintaining a heating rate of 10 °C min^−1^. Subsequently, the sample pan was placed into the PDSC cell, which was then closed and filled with dry air at a pressure of 200 psi (1378.95 kPa). The collection of data took place while the cell temperature was increased from room temperature (RT) to 300 °C with a rate of 10 °C min^−1^. Graphs of heat flow (W g^−1^) in relation to temperature were employed to determine the Oxidation Onset Temperature (OT, °C) and the Temperature of Signal Maxima (SM, °C) utilizing the designated software. Each sample underwent three distinct trials, and the average results, approximated to the closest tenth of a degree, are presented.

### 2.7. Viscosity, density, and viscosity index analysis

An SVM3001/G2 viscometer, paired with an Automatic Sample Changer (Xsample 530; Anton Paar GmbH, Graz, Austria), was employed to evaluate the densities and dynamic viscosity of WCOs and RSO at temperatures of 40 °C and 100 °C, as per ASTM D7042,^41^ and ASTM D4052,^42^ standards, respectively. The procedure entailed transferring 20 mL of each oil sample into a vial, which was then positioned inside the carousel of the automated sample changer. The apparatus automatically calculates and presents the kinematic viscosity at both 40 °C and 100 °C by leveraging the density and dynamic viscosity measurements obtained at these specific temperatures. In alignment with the ASTM D2270 standard, the viscosity index (VI) of the oil samples was subsequently determined automatically, utilizing the kinematic viscosity at 40 °C and 100 °C.^43^

### 2.8. Cloud point (CP)

The cloud point of oil samples was established in accordance with the ASTM D5773,^44^ standards using an automatic cloud/pour point apparatus (Phase Technology PSA-70x model). Before the analysis, samples were kept in the laboratory at a controlled RT of 22 ± 1 °C. An aliquot of 0.150 ± 0.005 mL of the sample was subsequently introduced into a chamber within the instrument, which features a reflective surface at the bottom, and was then closed. A vacuum was introduced into the sealed chamber to eliminate any ambient moisture. The oil samples underwent cooling at 1.5 ± 0.1 °C per minute. An internal light source continuously illuminated the interior of the chamber, which was directed at an angle onto the sample, while an optical detector positioned directly above the sample continuously monitored this process. In its liquid state, the light source passed through the sample and was reflected off the chamber's bottom. As the sample cooled and crystal formation commenced, the crystals caused the focused light to disperse, with a portion of it being directed toward the optical sensor located directly above the sample. The sample's cloud point, defined as the temperature at which crystal formation occurs, was established by monitoring the temperature of the sample when the directed light was reflected onto the optical sensor.

### 2.9. Pour point (PP)

The pour point (D5949) of the samples was measured right after the assessment of the cloud point (D5773). A surge of dry air was injected into the sample. During this process, the optical sensor identified a shift in optical scattering. The sample was uniformly cooled at a rate of 1.5 °C per minute, with dry air pulses introduced at integer intervals of every 3 °C. Upon freezing the sample, no significant alteration in the optical response was noted. The preceding measurement was subsequently identified as the sample's pour point. For instance, if an optical change is not detected when the dry air pulse is applied at −25 °C, the pour point is noted as −22 °C.

### 2.10. Tribological testing

All ball-on-flat type tribo-tests were conducted on a Universal mechanical testing platform (Paltro, UniTest 750) on a linear reciprocation motion module. Tests were conducted at ambient temperature under both electrified and unelectrified conditions. Normal Load of 20 N was applied with a stroke length of 10 mm at 5 Hz frequency, each test was run for 42 minutes, resulting in a total sliding distance of 250 meters. 10 mL of lubricant solution of each type was used for tribo tests, and two repeats of each test condition were performed to capture statistical significance of observed trends. The experimental conditions for both electrified and unelectrified cases were the same, except for the application of 1.5 A DC during electrified tests, as depicted in Table 1.

**Table 1:** Test conditions

Linear reciprocation motion	Unelectrified	Electrified
Load [N]	20	20
Frequency [Hz]	5	5
Stroke length [mm]	10	10
Test duration [minutes]	42	42
Sliding distance [m]	∼250	∼250
Amount of lubricant [ml]	10	10
Current [DC] [A]	0	1.5

**Fig. 1:**
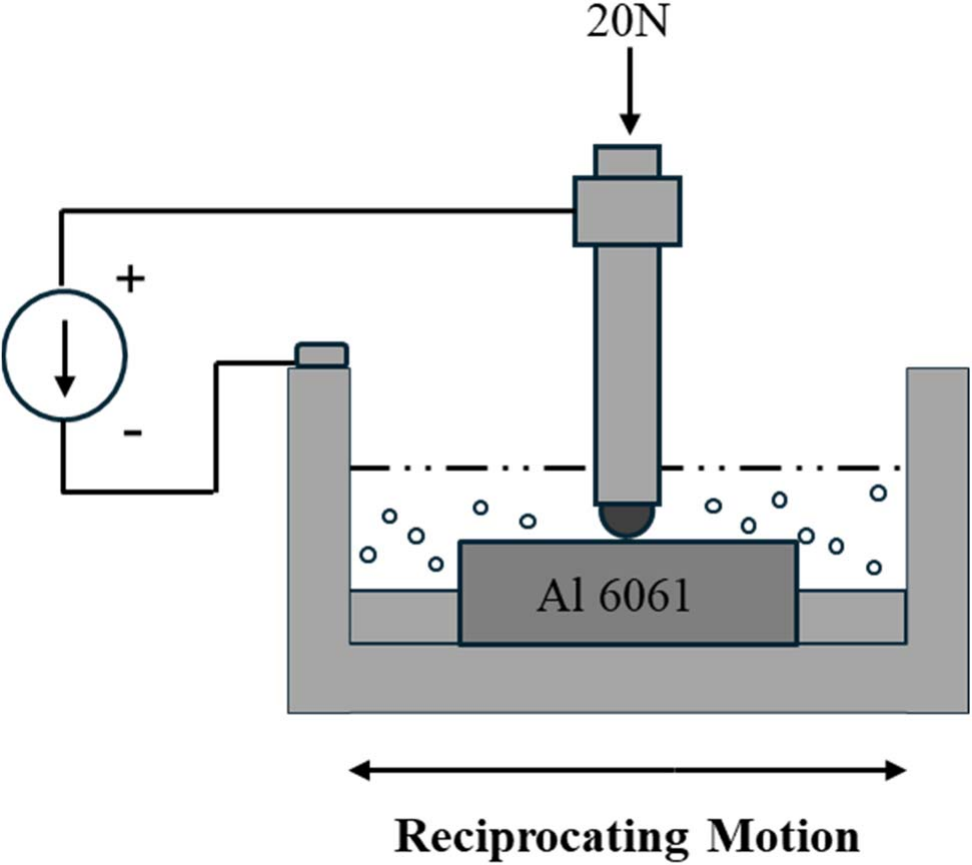
Schematic of the ball-on-flat reciprocating tribology test.

Since electric motors operate under both alternating and direct currents,^45^ direct current (DC) was used in this study to replicate the stray current. Previous studies reported that the stray current amplitudes from a 1.5 kW motor range between 0.2 to 1.4 A.^46^ Hence, a constant current of 1.5 A was chosen for this study. A programmable DC power supply (Siglent SP5161X) was used to deliver constant current. The positive terminal of the power supply was connected to the ball holder, and the negative terminal of the power supply was connected to the flat holder to ensure current flow through the lubricated contact. A schematic of the test configuration showing the electrical circuit is presented in Fig. 1.

Voltage and current data were simultaneously captured by the power supply throughout the tests. Post-surface characterization was conducted to evaluate the wear mechanisms and material transfer. A white light profilometer (NewView 9000, Zygo, Ametek) was used to assess the surface topography of both ball and flat specimens. Additionally, Scanning Electron Microscopy (SEM, Oxford Instruments) and Energy Dispersive Spectroscopy (EDS, Oxford Instruments) were used to examine the wear scars for signs of adhesion and material transfer. Raman spectroscopy analysis was conducted on the wear tracks, as it is considered one of the most widely used non-destructive characterization methods for analyzing tribo-films in the contact area (wear track), especially given the possibility of lubricant decomposition.^47^

## 3. Results and discussions

The results and discussions are broadly divided into three major sections: (a) structural analyses of lubricants, (b) evaluation of physico–chemical properties, and (c) assessment of tribological behavior.

### 3.1. Structural analysis of lubricants

#### 3.1.1. FTIR analysis

**Fig. 2:**
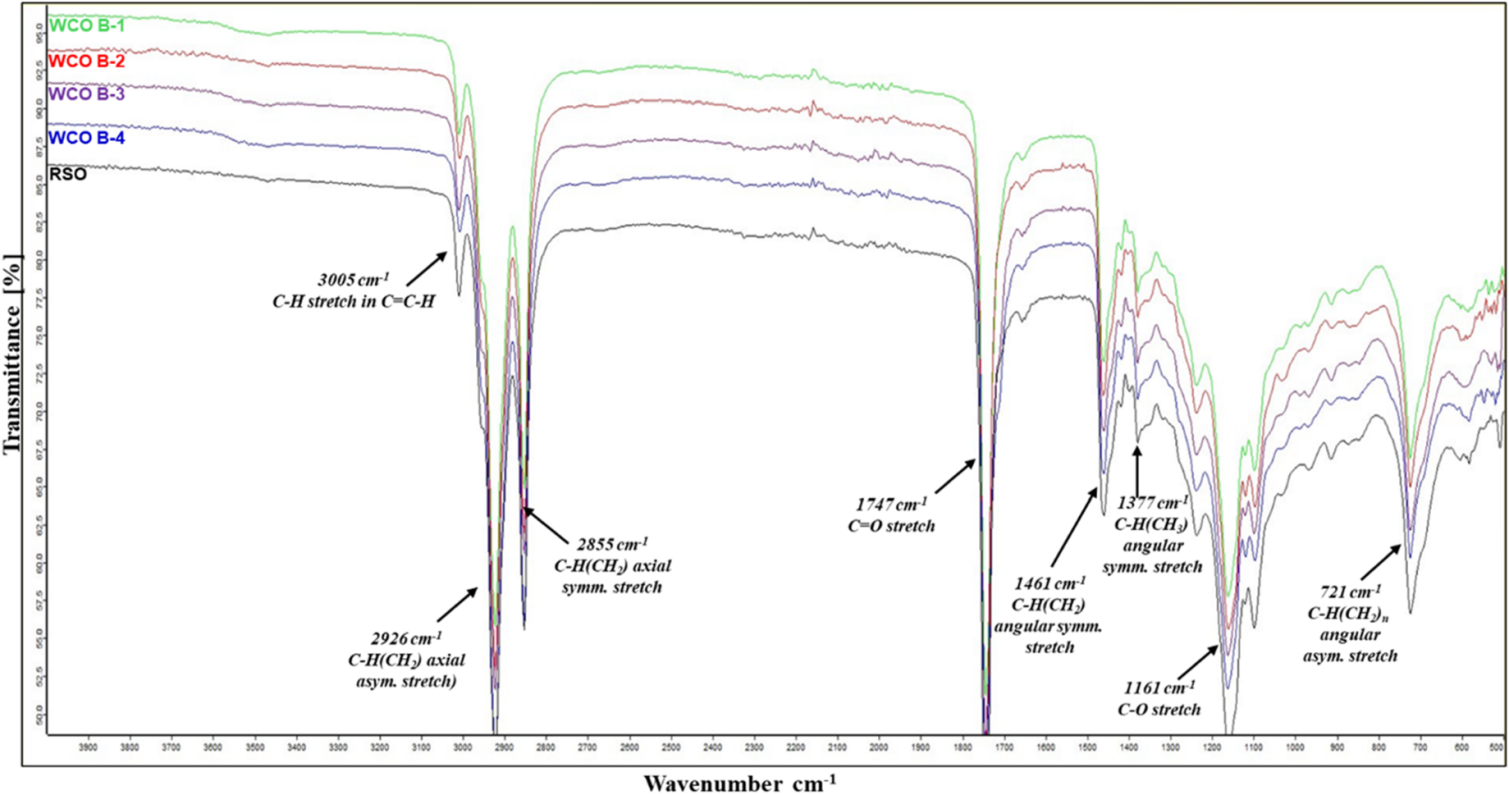
FTIR spectra of WCO B-1, WCO B-2, WCO B-3, WCO B-4, and RSO.

The FTIR spectra of WCO B-1, WCO B-2, WCO B-3, WCO B-4, and RSO are compared side by side in Fig. 2. The C=O stretching peak for the ester groups in both WCOs and RSO appears at 1747 cm^−1^.^48,49^ The lack of an absorption peak near 1710 cm^−1^ for C=O stretching for the carboxylic acid group suggests the absence of free fatty acids (FFA) in the oil samples. The C–O stretching bands corresponding to the ester groups in the WCOs and RSO are detected at 1161 cm^−1^.^50^ Additionally, the peaks at 3005 cm^−1^ signify C–H stretching on vinylic carbon (C=C–H)^51^ indicate that all WCOs and RSO possess unsaturated FA chains. Among other significant absorptions, the peaks at 2926, 2855, 1461, and 721 cm^−1^ correspond to axial asymmetric C–H(CH_2_), axial symmetric C–H(CH_2_), angular symmetric C–H(CH_2_), and angular asymmetric C–H(CH_2_) stretches, respectively. The spectral similarity noted between RSO and WCOs suggests that all WCOs maintain their triacylglycerol structural integrity.

#### 3.1.2. NMR analysis

**Fig. 3:**
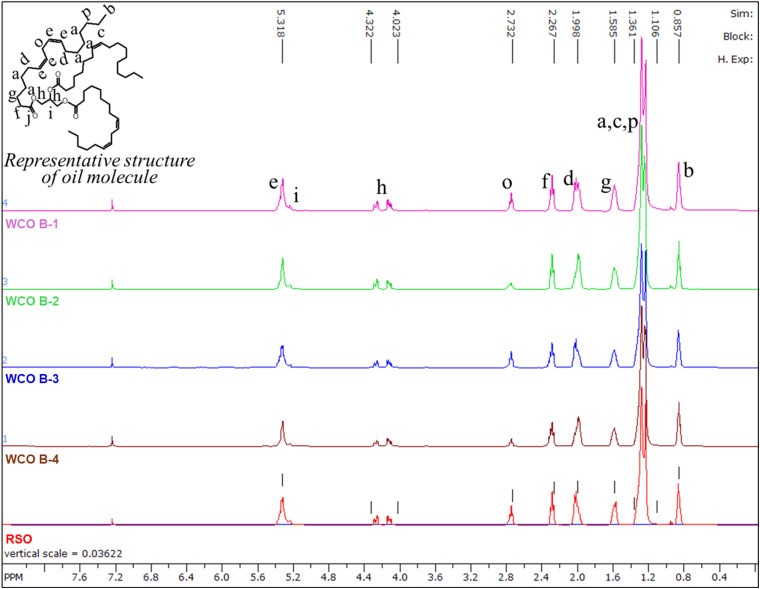
The ^1^H NMR spectra of WCO B-1, WCO B-2, WCO B-3, WCO B-4, and RSO. Spectral peaks are designated with letters corresponding to their respective protons.

**Fig. 4:**
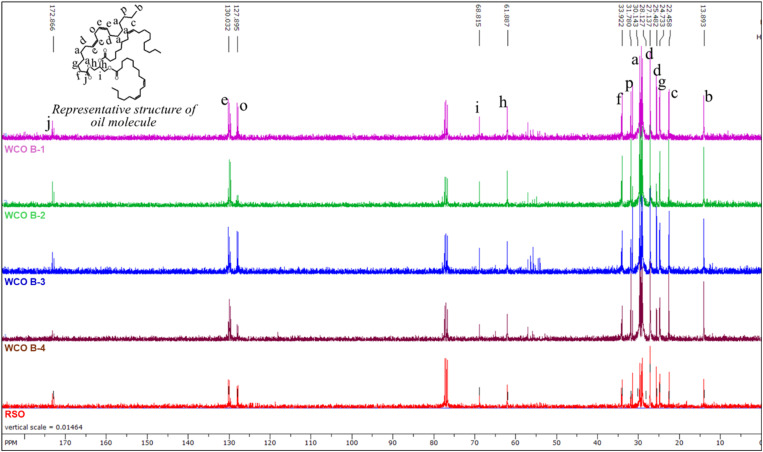
The ^13^C NMR spectra of WCO B-1, WCO B-2, WCO B-3, WCO B-4, and RSO. Spectral peaks are designated with letters corresponding to their respective protons.

The ^1^H and ^13^C NMR spectra of oils are illustrated in Fig. 3 and 4, respectively. As anticipated, the ^1^H and ^13^C spectra exhibit similarities across all waste cooking oils (WCOs) and regular soybean oil (RSO).

In ^1^H NMR spectra (Fig. 3), the peak observed at 0.85 ppm is consistent across all oils, corresponding to the terminal –CH_3_ protons found in the fatty acid chains of triglyceride molecules. The peaks at 1.99, 2.73, and 5.31 ppm represent the allylic, bis-allylic, and vinylic protons, respectively, indicating the existence of unsaturated fatty acid chains in all WCOs and RSO. Notably, the relative peak areas for bis-allylic protons (2.73 ppm) are comparatively larger in WCO B-1, WCO B-3, and RSO than in WCO B-2 and WCO B-4, suggesting that polyunsaturated fatty acid chains are more prevalent in WCO B-1, WCO B-3, and RSO compared to WCO B-2 and WCO B-4. The peaks ranging from 4.02 to 4.32 ppm are present in all samples, representing the protons of the glycerol backbone, thereby affirming that the backbone stays preserved across all WCOs. All other peaks have been identified according to the corresponding protons in the representative structure of the oil molecules.

The ^13^C spectra (Fig. 4) for all the oils are similar, as expected. Each peak has been labeled with a letter corresponding to the equivalent carbon. The peak representing the terminal carbon of fatty acid chains is located at the highest upfield position of the spectrum (13.8 ppm). The peaks at 61.8 and 68.8 ppm correspond to the carbons in the glycerol backbone of the oils. The peaks at 127.8 and 130.0 ppm represent the bis-allylic and vinylic carbons, respectively. A comparative analysis of peak intensity for bis-allylic carbons clearly shows that WCO B-1, WCO B-3, and RSO contain a higher concentration of polyunsaturated chains than WCO B-2 and WCO B-4. The ester carbonyl carbons of the oils are commonly indicated by the peak at 172.8 ppm. The absence of a peak near 180 ppm indicates that FFA are either not present in the oil samples or are below the detection limit using this technique.^52,53^

#### 3.1.3. GC-MS analysis

**Fig. 5:**
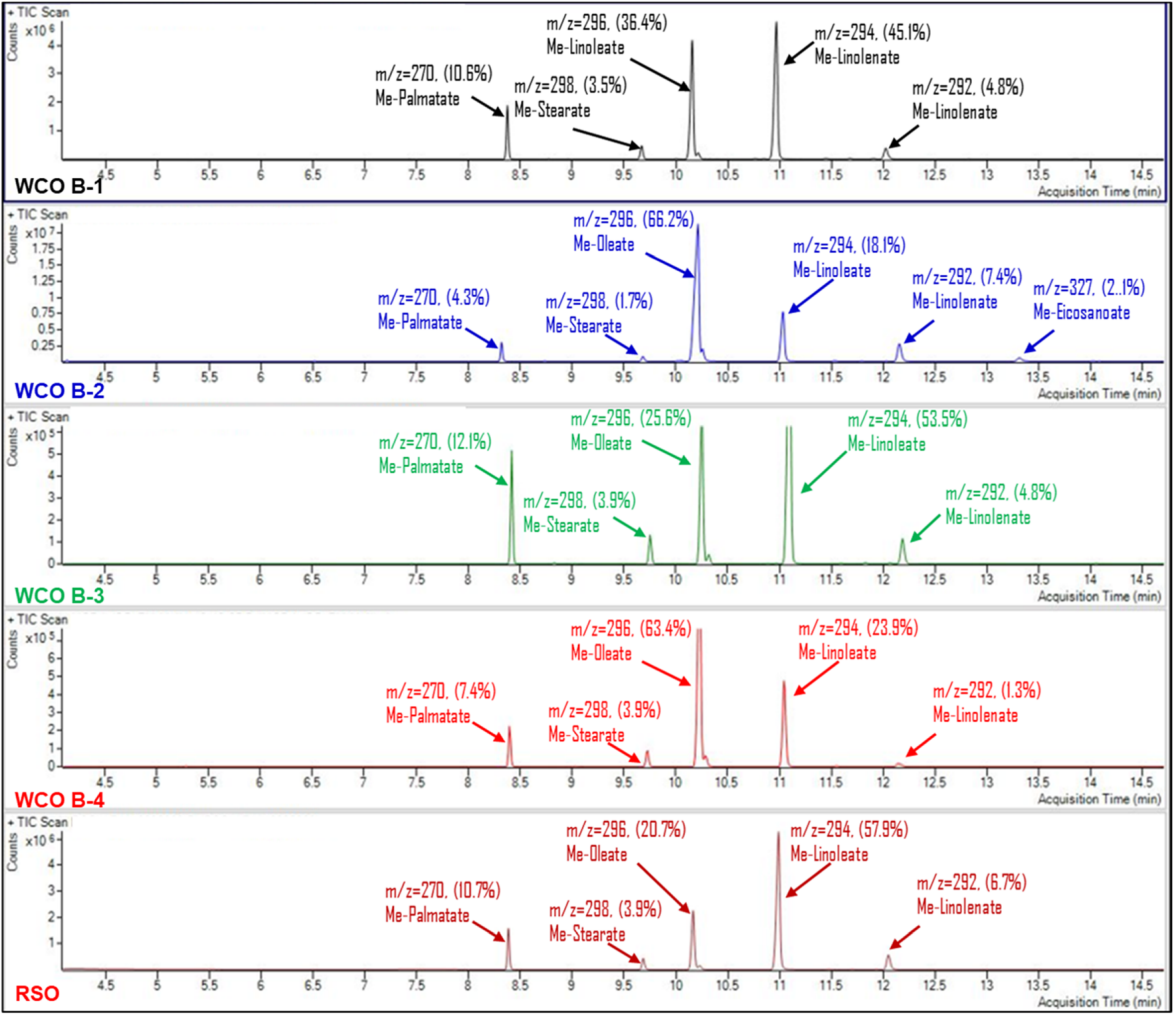
GC-MS total ion count in relation to retention time for FAMEs of WCO B-1, WCO B-2, WCO B-3, WCO B-4, and RSO. The peaks are designated with the corresponding FAME molecular weight and relative area percentage.

The GC-MS data presented in Fig. 5 illustrate the fatty acid methyl ester (FAME) profiles for all WCOs and RSO. Five fatty acid chains, namely palmitic, stearic, oleic, linoleic, and linolenic acids, are identified as common across all oil samples. Notably, a minor presence of eicosanoic acid (2.1%) is observed in the WCO B-2 sample. The FAME compositions for the various WCOs and RSO are detailed in Table 2.

**Table 2:** Composition of FAMEs of WCO B-1, WCO B-2, WCO B-3, WCO B-4 and RSO

Biolubricant base oil	Palmitic acid	Stearic acid	Oleic acid	Linoleic acid	Linolenic acid	Eicosanoic acid
WCO B-1	10.6%	3.5%	36.4%	45.1%	4.2%	
WCO B-2	4.3%	1.7%	66.2%	18.1%	7.4%	2.1%
WCO B-3	12.1%	3.9%	25.6%	53.5%	4.8%	
WCO B-4	7.4%	3.9%	63.4%	23.9%	1.3	
RSO	10.7%	3.9%	20.7%	57.9%	6.7%	

The total relative area percentages of unsaturated fatty acids in WCO B-1, WCO B-2, WCO B-3, WCO B-4, and RSO are recorded as 85.7%, 91.7%, 83.9%, 88.6%, and 85.3%, respectively. The relative percentages of polyunsaturated fatty acid chains are calculated to be 49.3% for WCO B-1, 25.5% for WCO B-2, 58.3% for WCO B-3, 25.2% for WCO B-4, and 64.6% for RSO. This data suggests that WCO B-1, WCO B-3, and RSO possess a higher proportion of polyunsaturated fatty acid chains compared to WCO B-2 and WCO B-4. These findings align with the results obtained from ^1^H and ^13^C NMR analyses.

#### 3.1.4. CMS analysis

**Fig. 6:**
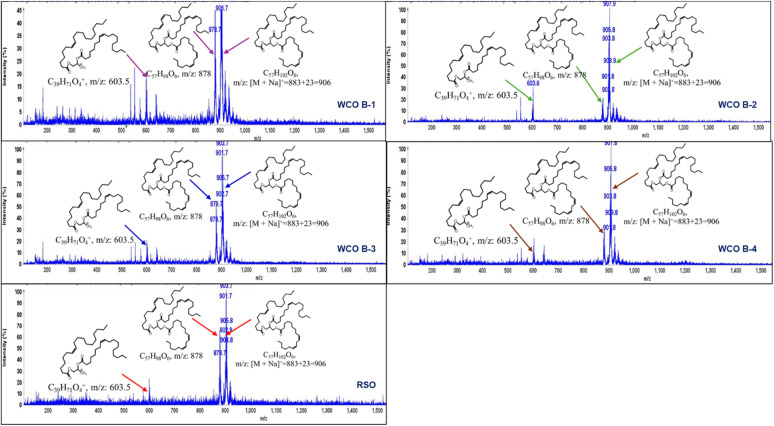
CMS analysis of WCO B-1, WCO B-2, WCO B-3, WCO B-4 and RSO with their fragmented ions.

Fig. 6 illustrates the analytical spectra obtained from CMS for the WCOs and RSO utilizing an ESI ion source. All oil samples are commonly represented by three primary ions at 906, 878, and 603.6 *m*/*z*. The molecular mass of 906 *m*/*z* represents an adduct ion formed by the combination of the oil-derived triglyceride molecule (883 *m*/*z*) with a sodium ion (Na^+^ = 23 *m*/*z*) originating from the glassware. The molecular mass of 878 *m*/*z* is indicative of a distinct composition of the whole triglyceride molecular ion of the oil, without the formation of any adduct product. The fragment at *m*/*z* = 603.6 denotes a diglyceride molecule. However, the broad peaks observed in the spectra suggest either the presence of the same molecular formula of oil-derived ions with varying isotopic distributions or oil-derived ions with slightly different compositions in their fatty acid chains. This data unequivocally affirms the integrity of the chemical structure of all WCOs and RSO.

### 3.2. Physico–chemical properties of the WCOs and RSO

#### 3.2.1. Density

The densities of WCOs are evaluated in comparison to those of RSO. The data presented in Table 3 indicate that there are no significant variations in density among the oils tested at either 40 or 100 °C. Both WCO B-3 and WCO B-4 demonstrate a marginally higher density than RSO at 40 °C, and a similar pattern is observed at 100 °C. Conversely, WCO B-1, WCO B-2, and RSO exhibit nearly identical densities at both temperature points. This minor variation in densities between the WCOs and RSO can be ascribed to the combined influence of dissolved food residues and the *cis*/*trans* stereoisomeric effect. The density of oils is primarily determined by how tightly their fatty acid chains are packed; linear *trans*-unsaturated chains offer greater compactness compared to bent *cis*-unsaturated chains.^37^ In RSO, the fatty acid chains are predominantly in the cis form, whereas the C=C bonds in WCOs are thought to have partially converted to *trans* isomers due to exposure to high temperatures and prolonged heat of cooking.^54^ As anticipated, the density of all oils decreases with a rise in temperature.

**Table 3:** Comparison of the physicochemical properties of WCOs and RSO^a^

	Temp°C	WCO B-1	WCO B-2	WCO B-3	WCO B-4	RSO
Density (g cm^−3^)	40	0.9089 ± 0.0	0.9047 ± 0.0	0.9102 ± 0.0	0.9102 ± 0.0	0.9066 ± 0.0
	100	0.8689 ± 0.0	0.8650 ± 0.0	0.8704 ± 0.0	0.8706 ± 0.0	0.8668 ± 0.0
Kinematic viscosity (mm^2^ s^−1^)	40	39.7 ± 2.5	37.7 ± 0.0	37.5 ± 0.0	51.8 ± 0.0	30.69 ± 0.0
	100	8.7 ± 0.7	8.5 ± 0.0	8.3 ± 0.0	10.1 ± 0.0	7.53 ± 0.0
Dynamic viscosity (mPa s^−1^)	40	36.1 ± 1.8	34.1 ± 0.0	34.17 ± 0.0	47.10 ± 0.0	27.82 ± 0.0
	100	7.55 ± 0.6	7.35 ± 0.0	7.23 ± 0.0	8.82 ± 0.0	6.52 ± 0.0
Viscosity index		206.3 ± 0.2	212.88	206.18 ± 0.0	187.76 ± 0.0	228.4 ± 0.1
Oxidative onset temp, OT (°C)		131.8 ± 0.1	184.0 ± 0.4	141.3 ± 0.3	136.4 ± 0.4	175.7 ± 2.0
Oxidative peak temp, PT (°C)		173.6 ± 0.6	200.4 ± 3.2	183.5 ± 2.4	191.0 ± 2.5	189.8 ± 1.8
PP (°C)		−6.0 ± 0.0	−18.0 ± 0.0	−6.0 ± 0.0	−6.0 ± 0.0	−15 ± 0.2
CP (°C)		−3.5 ± 0.0	−13.5 ± 0.2	−0.9 ± 0.1	0.65 ± 0.2	−8.1 ± 0.2
HFRR (µm)		240				290

a Every property value denotes an average calculated from three individual measurements.

#### 3.2.2. Kinematic viscosity and viscosity index

Kinematic viscosity denotes fluid's ability to counter internal flow when influenced by gravitational forces. The measurement of kinematic viscosity entails determining the time, in seconds, required for a defined volume of fluid to traverse a specific distance under the influence of gravity in a viscometer that has been meticulously calibrated for temperature regulation. Conversely, the viscosity index (VI) serves as a metric that describes how a fluid's thickness varies with temperature fluctuations. A high VI indicates that a fluid's viscosity is less prone to change across a wide temperature spectrum, thereby rendering it more stable across diverse thermal conditions. Table 3 presents a comparative analysis of the kinematic viscosity and viscosity index (VI) of waste cooking oils (WCOs) and refined sunflower oil (RSO).

At 40 °C, all WCOs demonstrate higher kinematic and dynamic viscosities compared to RSO (Table 3). As the temperature increases, the viscosities of all oils decrease. The viscosity diminishes exponentially with rising temperature. The higher viscosity of WCOs compared to RSO is likely attributable to the presence of dissolved food residues and polymerization that may have happened during cooking in WCOs.^55^ Among the WCOs, WCO B-4 exhibits a significantly higher viscosity than the other three oils. The number of double bonds present in the triglyceride molecules of the WCOs can explain this observation. Previous research has indicated that viscosity decreases as the number of double bonds within the molecule increases.^56^ The fatty acid composition of the oils, as shown in Table 2, reveals that the calculated number of C=C double bonds per triglyceride molecule in WCO B-1, WCO B-2, WCO B-3, and WCO B-4 are 4.17, 3.73, 4.4, and 3.43, respectively. WCO B-4 possesses the lowest number of double bonds per molecule, which contributes to its higher viscosity compared to the other WCOs. The viscosity index (VI) of oils, as per the ASTM D2270 standard, was subsequently assessed automatically utilizing the kinematic viscosity measurements at 40 °C and 100 °C.^38^Table 3 further illustrates that the viscosity index follows the order RSO > WCO B-2 > WCO B-1 > WCO B-3 > WCO B-4.

#### 3.2.3. Oxidation stability

The oxidation stability of WCO and RSO was assessed using Pressure Differential Scanning Calorimetry (PDSC), Table 3 provides a detailed account of the temperatures at which onset oxidation (OT) and peak oxidation (PT) took place. A higher OT and PT value signifies greater resistance to oxidation. In comparison, WCO B-2 demonstrates higher PT and OT values relative to RSO. The oxidative stability of vegetable oils is affected by their degree of unsaturation; oils with a higher degree of unsaturation tend to have diminished oxidation stability.^57^ As previously noted, WCO B-2 contains an average of 3.73 carbon–carbon double bonds (C=C) per triglyceride molecule, whereas RSO has 4.7 C=C per triglyceride molecule. This accounts for the superior stability of WCO B-2. Additionally, the presence of various dissolved food residues in all WCO samples impacts their PT values, making it challenging to elucidate their stability based solely on structural characteristics.

#### 3.2.4. Cloud point and pour point

The cloud point, often abbreviated as CP, signifies the temperature at which oil starts to exhibit a cloudy appearance as it cools. Conversely, the temperature at which oil ceases to flow is referred to as the pour point (PP). As illustrated in Table 3, RSO and WCO B-2 demonstrate lower CP and PP values when compared to the other three WCOs. Between RSO and WCO B-2, it is WCO B-2 that presents the lower CP and PP. This phenomenon is likely attributable to WCO B-2 possessing the lowest proportion of saturated fatty acid chains in its molecular structure (8.3%) compared to RSO (14.7%) and other WCOs. Linear saturated chains can be more easily stacked than their unsaturated counterparts.^48^ Oils that contain a lower amounts of saturated and higher amounts of unsaturated fatty acid chains are typically associated with lower pour points.^58^

### 3.3. Assessment of tribological behavior

#### 3.3.1. Coefficient of friction analysis, wear analysis

**Fig. 7:**
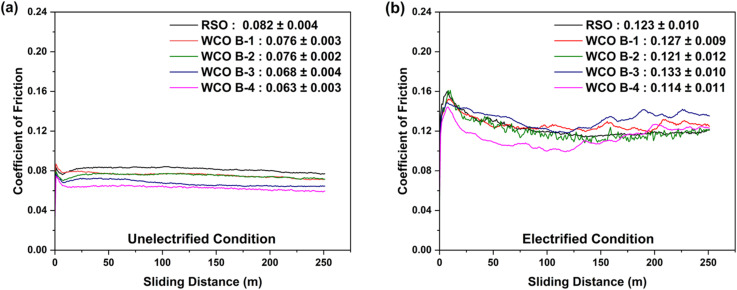
Average coefficient of friction trends (a) unelectrified (b) electrified.

Fig. 7 shows the variation in the average coefficient of friction (CoF) trend as a function of sliding distance under (a) unelectrified and (b) electrified conditions while lubricated *via* RSO and WCO batches B-1 to B-4. Average coefficient of friction (CoF) trends were derived from repeated trials to evaluate the statistical significance of measured numbers. Under unelectrified conditions, all lubricant samples demonstrated relatively steady CoF after an initial short running-in period. RSO exhibited the highest mean CoF of 0.082 ± 0.004, followed by WCO B-1 and B-2, both at 0.076, with slightly different standard deviations of ± 0.003 and ± 0.002, respectively. WCO B-3 and B-4 showed lower CoF values of 0.068 ± 0.004 and 0.063 ± 0.003, indicating improved lubricity. Among all, WCO B-4 resulted in the lowest CoF, suggesting it may provide better tribo-film formation or surface protection in unelectrified conditions. One of the reasons could be the higher viscosity of WCO B-4.

**Fig. 8:**
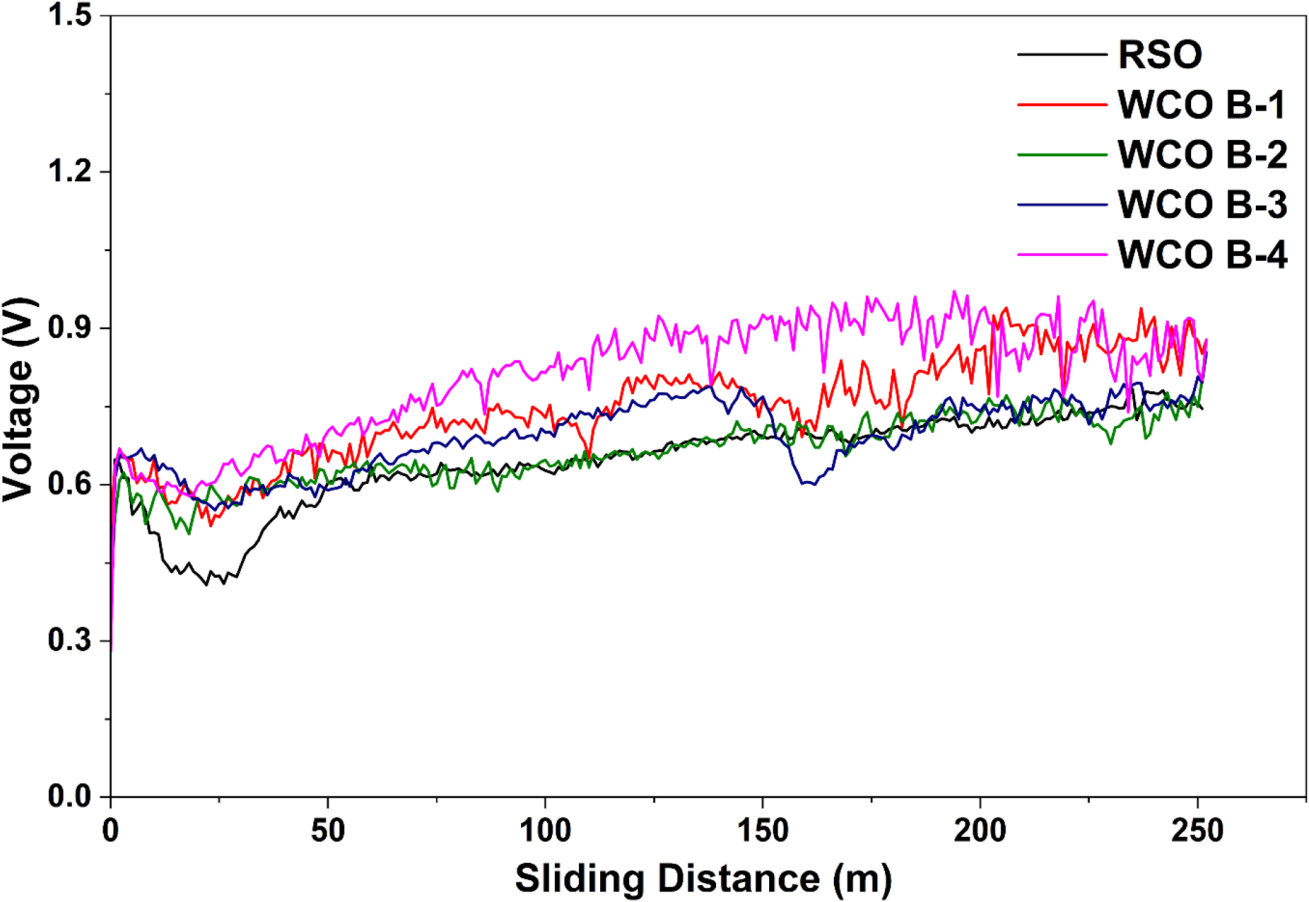
Evolution of recorded voltage as a function of sliding distance under electrified condition.

Previous studies have associated molecular structures of fatty acids strongly influencing frictional behavior. In particular, oils with high oleic acid content tend to yield lower CoF, whereas higher linolenic acid is associated with increased friction, due to differences in packing density and film-forming ability of monolayers.^59^ In contrast, CoF increased significantly for all samples under electrification (Fig. 7b). RSO exhibited a mean CoF of 0.123 ± 0.010, while WCO B-1 and B-2 were close at 0.127 ± 0.009 and 0.121 ± 0.012, respectively. WCO B-3 demonstrated the highest CoF of 0.133 ± 0.010, whereas WCO B-4 again showed the lowest value of 0.114 ± 0.011. A similar increase in CoF was reported in the study by Oscar *et al.*, where gear oil and ATF III exhibited an increased coefficient of friction under electrified conditions primarily due to accelerated oxidation of steel surfaces, which generated oxide particles that acted as abrasives.^20^ It is worth noting that WCO B-4 showed a significant reduction in friction as compared to the rest of the lubricant samples until around 125 m sliding distance, and beyond that, the CoF started increasing under electrified conditions. We believe the addition of a suitable lubricant additive package and chemical modification can help resolve this challenge. Overall, WCO B-4 showed a notable reduction in friction as compared to RSO in both electrified and non-electrified conditions. As depicted in Fig. 8, an inverse relationship between voltage and coefficient of friction. If we compare the progression of the friction coefficient (Fig. 7b) and the recorded voltage (Fig. 8) as a function of sliding distance, it can be found that a decrease in CoF was associated with an increase in the voltage signal, and *vice versa*. This observation aligns with the findings of Mohamed M. K.,^60^ where oil free from polymeric additives displayed a decreased CoF with increasing voltage. Since the current in this study was kept constant while the voltage varied, a similar trend can be observed here, where the CoF decreased as the voltage increased.

**Fig. 9:**
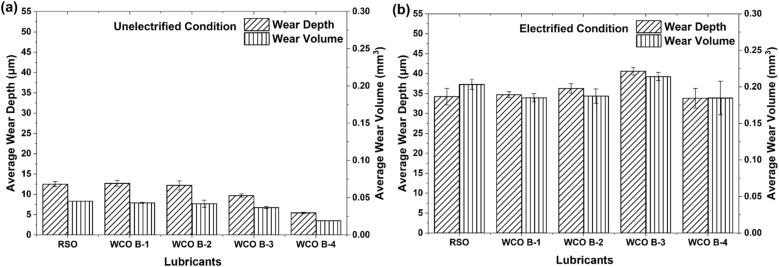
Average wear depth and wear volume of Al flats (a) unelectrified (b) electrified.

Average wear track depths were measured using the white light interferometry technique on aluminum flat samples tested under both electrified and unelectrified conditions, and the corresponding wear volumes were presented in Fig. 9. A good synergy between average wear track depth and volumes on samples lubricated under different lubricants can be observed. WCO B-4 showed the shallowest wear depth of 5.44 ± 0.24 µm at the center and the lowest average wear volume of 0.019 ± 0.001 mm^3^, while RSO recorded a wear depth of 12.50 ± 0.64 µm and volume of 0.045 ± 0.001 mm^3^. It should be noted that WCO B-4 possessed significantly higher viscosity than the rest of the lubricant types, which could have resulted in the lowest friction coefficient under both electrified and non-electrified conditions.

**Fig. 10:**
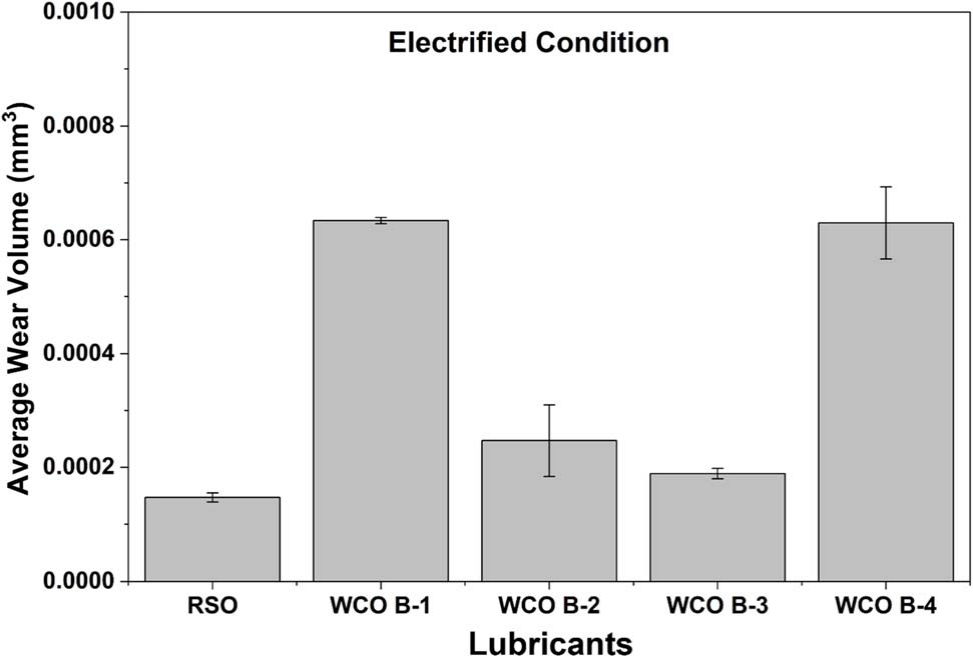
Average wear volume of 52 100 balls.

This strong alignment between lower CoF and reduced wear suggests effective tribo-film formation for certain WCO batches, particularly WCO B-4, in unelectrified conditions. Electrification resulted in higher wear across all lubricants, similar to the previous observation on CoF numbers. WCO B-3 produced the deepest wear track of 40.60 ± 0.93 µm and the largest wear volume 0.214 ± 0.006 mm^3^, while WCO B-4 maintained relatively lower values of wear track depth and volume (33.84 ± 2.47 µm and 0.185 ± 0.023 mm^3^ respectively) were observed. The lowest wear resistance of WCO B-3 under electrified conditions is consistent with the highest friction coefficient. The potential reason behind the poor tribological behavior of WCO B-3, along with similar performance observed in RSO and WCO B-1 under electrified conditions, can be attributed to (1) their higher number of C=C double bonds per triglyceride molecule that makes the lubricant more prone to oxidation^61^ and (2) the presence of food particles/polar impurities. The difference between wear volumes for RSO and WCO B-4 got reduced in the electrified condition, unlike the unelectrified case. Inability to have surface protection capability beyond a certain sliding distance (∼125 m) played a major role in WCO B-4 lubricant's electrified testing. The reduced CoF trend was consistent for the whole 250 m sliding distance in unelectrified testing. The average wear volumes of the 52 100 steel balls used as counter bodies under electrified conditions were measured and presented in Fig. 10; the wear scars were almost invisible for unelectrified samples. WCO B-3 exhibited the lowest ball wear volume of all WCO samples, at 0.000189 ± 0.000009 mm^3^, followed by WCO B-2 at 0.000247 ± 0.000063 mm^3^. In contrast, WCO B-1 showed the highest wear at 0.000634 ± 0.000005 mm^3^. RSO recorded a relatively lower value of 0.000147 ± 0.000008 mm^3^. This suggests that there was an inverse relationship between the stability of ball and flat materials under electrified conditions. AISI 52100 steel was significantly stable as compared to aluminum flat samples while WCO B-3 lubricated tests under electrified condition. This resulted in high friction and increased wear on flat samples, but the ball wear was minimal compared to other samples.

#### 3.3.2. Wear morphology and mechanisms under unelectrified conditions

**Fig. 11:**
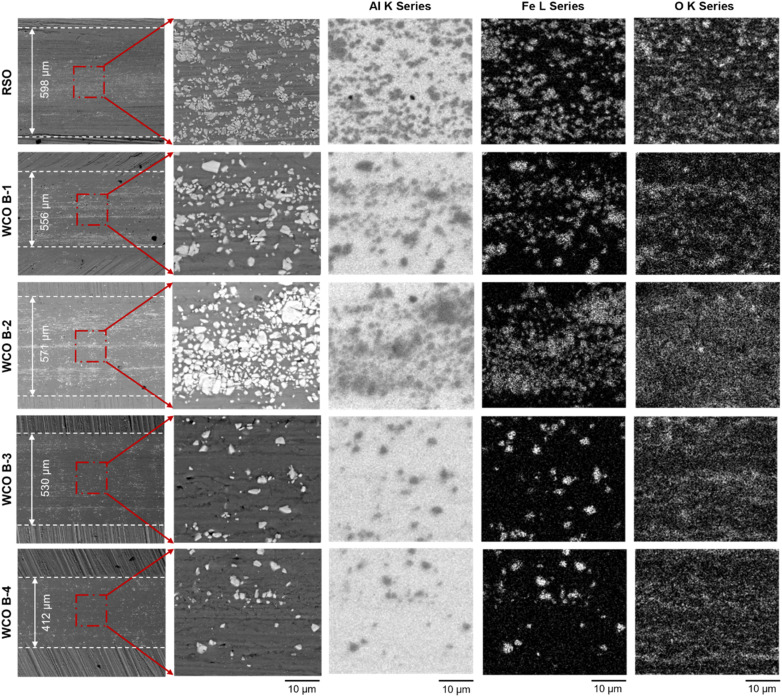
SEM EDS-elemental analysis of test samples experimented using different lubricants under unelectrified condition.

Fig. 11 presents a comparative analysis of secondary electron micrographs and key elemental mapped data captured using EDS from the center of the wear tracks experimented under unelectrified conditions. The secondary electron images were captured using both low and high magnification to gain a good understanding of the overall wear track morphology and to obtain deeper insights, along with corresponding elemental maps (high magnification) for aluminum (Al), iron (Fe), and oxygen (O). The RSO sample exhibited a relatively broad wear track width (∼598 µm) as compared to that of WCO B-1 to B-4 samples (ranging from ∼571 µm to 412 µm), with B-4 exhibiting the smallest wear scar width. The EDS elemental maps offer insight into material transfer mechanisms and surface reactions during tribo-testing. The base material of the flat specimen was aluminum, while the counter body used was a steel ball. Therefore, Al maps highlight the substrate surface, whereas Fe maps reveal material transfer from the steel ball to the flat sample during experiments. It is worth noting that the Fe and Al contrast regions are complementary to each other, but oxygen signals were captured from both Al and Fe-rich regions. This shows both Al wear track and transferred Fe got oxidized during or after experiments. Additionally, the ball-to-flat material transfer was significantly high for RSO, WCO B-1, and B-2 lubricated conditions, and this was reduced significantly in WCO B-3 and B-4.

#### 3.3.3. Wear morphology and mechanisms under electrified conditions

**Fig. 12:**
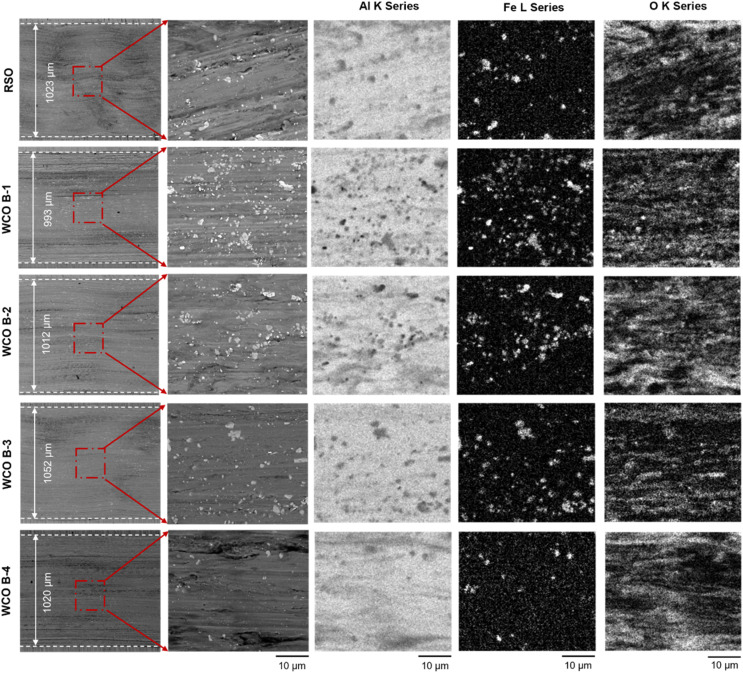
SEM EDS-elemental analysis of test samples experimented using different lubricants under electrified condition.

Fig. 12 presents SEM-EDS analyses of wear tracks for the samples lubricated with RSO and four WCO batches (B-1 through B-4) under electrified conditions. The WCO B-3 sample exhibited the widest wear scar (∼1052 µm) against all other test conditions. Compared to unelectrified conditions, the wear scar widths under electrification increased significantly for all lubricants, which is consistent with observed trends in friction and wear behaviors. The WCO-lubricated samples exhibited wear widths ranging from ∼993 µm (B-1) to ∼1052 µm (B-3), with B-1 having the narrowest wear region and B-3 the widest. The high-magnification secondary electron micrographs reveal groove widening, lower ball to flat material transfer in all electrified testing experiments, as compared to those of unelectrified cases, along with spatial variations in intensity and distribution of transferred material within wear track regions. WCO B-4 and RSO exhibited low Fe signals, suggesting reduced adhesive wear. There was no major difference in the spatial variations of the intensity of oxygen inside wear track regions among different samples. In the electrified wear tracks, the grooves appeared deeper, and the wear mechanism was a combination of abrasive and adhesive wear with increased abrasive wear percentage as compared to the unelectrified case. This observation aligns with the study by Leonardo *et al.*, where the wear mechanisms of gear oil, ATF, and mineral oil changed significantly under electrified conditions, primarily due to oxidation of steel surfaces, which generated oxide debris and led to abrasive wear.^21^

#### 3.3.4. Raman spectroscopy analysis

**Fig. 13:**
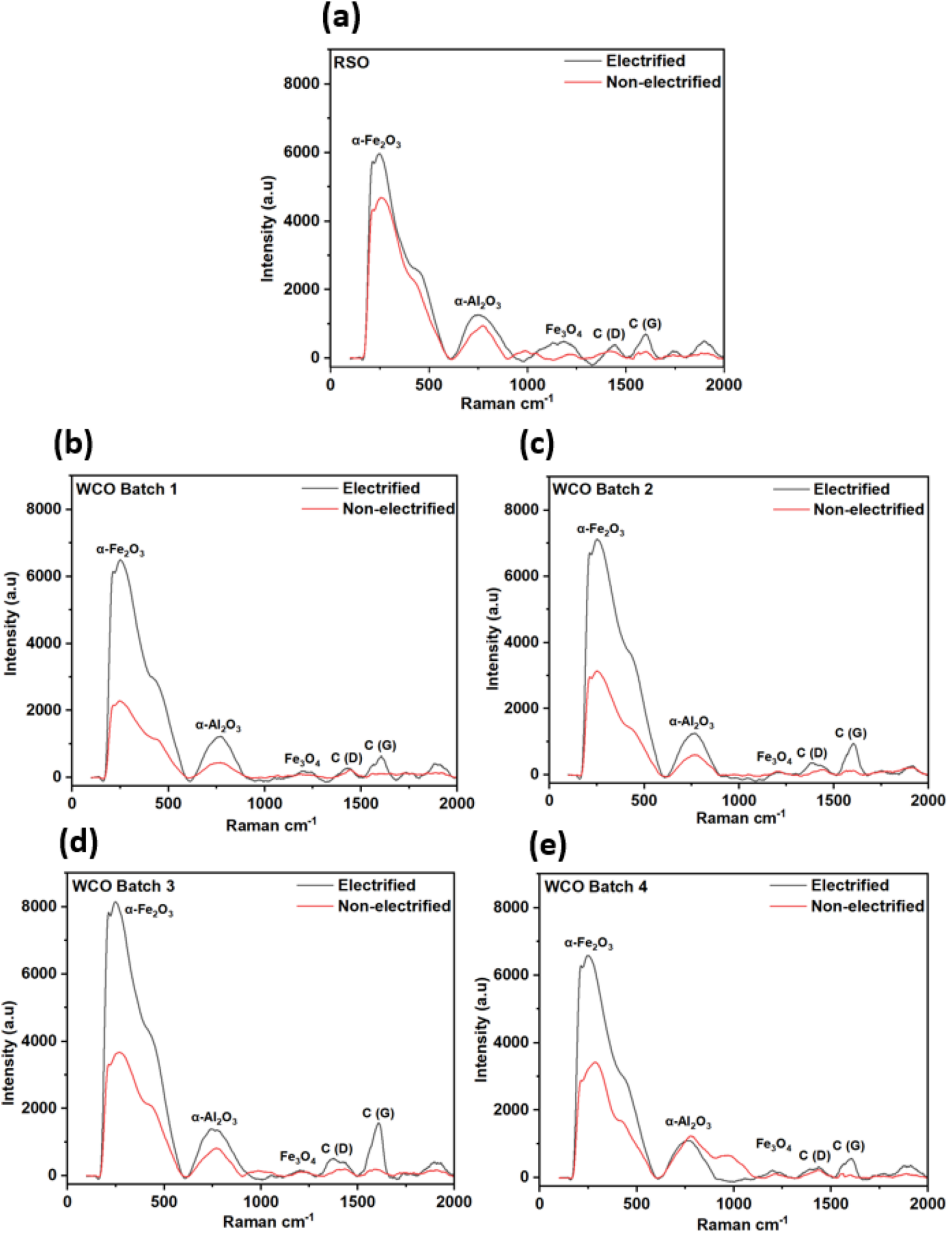
Raman spectroscopy analysis of selected lubricants under electrified and non-electrified conditions, (a) RSO, (b–e) WCO batch 1–4.

The point Raman analysis was conducted utilizing Renishaw Dispersive-Raman with a 588 nm solid state laser focusing inside the wear track regions of the aluminum flat sample from lubricated wear tests for both non-electrified and electrified conditions and presented in Fig. 13. Although the distinctive Raman peaks of hematite (α-Fe_2_O_3_), magnetite (Fe_3_O_4_), corundum (α-Al_2_O_3_), D (amorphous), and G (graphitized) bands of carbon were found in all the test samples regardless of conditions, the Raman spectra of the five different lubricating oils revealed significant differences in oxide formation under non-electrified and electrified conditions. The characteristic Raman peak of α-Fe_2_O_3_ and Fe_3_O_4,_ which appeared around 222 cm^−1^ and 1224 cm^−1^, indicated the oxidation of the steel counter body, which got transferred to the wear tracks on the aluminum flat^47,62^ samples. This confirms a continuous material transfer mechanism from the harder counter body (steel ball) to the generated tribo-layers by forming softer oxides. Additionally, the presence of α-Al_2_O_3_ peaks appeared around 750 cm^−1^, confirming that the oxidative reactions involved the bare flat surface.^63^ Moreover, the detection of the amorphous carbon band around 1360 cm^−1^ (D-band) and 1600 cm^−1^ (G-band), corresponding to the disordered (amorphous) carbon, indicates the decomposition of the carbonaceous component of the lubricants during the sliding tests.^64^ The reason behind the formation of those oxides and decomposed carbon could result from the increased surface energy, possibly originating from several factors associated with the rubbing surfaces.^65^ Highlighting the catalytic chemical reaction induced by mechanical forces, tribo-emission could be related to increased surface energy, where the mechanical forces transfer energy to a flux of electrons, activating atoms for reactions.^66^ Apart from that, the shear stress originated from the contact pressure could also be directly responsible for the tribo-catalytic reactions between contacting surfaces, further enhancing the formation of the tribo-layer. Although those phenomena pose a comparable effect within all the test conditions and lubricant types, the primary cause for the distinctly higher intensities in the electrified tests compared to the non-electrified ones is most likely associated with the electric potential presented between the flat and counter body, creating a contact resistance and sufficiently influencing the extent of tribo-film formation. From Fig. 13, it is evident that for all lubricants, the oxide-enriched tribofilms produced during the electrified wear tests were much more potent as compared to their corresponding non-electrified conditions. It can be speculated that the maximum wear for each case occurred on the flat aluminum sample (cathode) and hard oxide film on the counter steel ball (anode), eventually supplementing the overall wear phenomena.^67^Fig. 13 depicts a significant difference in the characteristics of the tribo-film between the WCOs (1–4) and the RSO. While there were substantial differences between the intensities of the oxides between the electrified and non-electrified conditions, the peak corresponding to hematite was significantly increased in the RSO lubricated case under non-electrified conditions (Fig. 13a) which reduced the intensity differences between electrified and non-electrified conditions. It could be speculated that the introduction of the electric potential affected the tribo-film formation in the WCO more prominently than that for the RSO lubricants and predominantly increased the graphitized carbon regardless of the lubricant types. Additionally, the formation of the Fe_2_O_3_ might have promoted further lubricant degradation, and excessive Fe_2_O_3_ particles increased the overall abrasive wear.^62^ The deeper grooves that were observed in Fig. 12 can be attributed to the formation of aluminum oxide and ferrous oxides acting as abrasive particles and deepening the wear track, since they are significantly harder than aluminum.^68^ Higher peak intensity of Fe_2_O_3_ seems to have direct relation with the highest wear observed for the corresponding flat samples in both test conditions (electrified and non-electrified) for WCO B-3 lubricating oil case. On the other hand, the higher intensity of the graphite peak in WCO B-3 lubricant for the electrified condition can be associated with higher breakdown tendency of the lubricating oil under electric potential and consequently that resulted increased wear on corresponding aluminum flat sample.

## 4. Conclusions

The chemical, physicochemical, and tribological properties of four batches of waste cooking oil (WCO) in comparison with regular soybean oil (RSO) under both electrified and unelectrified sliding conditions were investigated to evaluate their potential as sustainable biobased lubricant for electric vehicle (EV) drivetrains.

• Chemical and structural analyses using FTIR, ^1^H/^13^C NMR, GC-MS, and CMS confirmed triglyceride backbone integrity in all samples. Variations in fatty acid composition influenced viscosity and oxidation stability. WCO B-4, with the lowest unsaturation of 3.43, C=C/triglyceride showed the highest viscosity. WCO B-2 with high oleic acid content of 66.2% in combination with lower unsaturation of 3.73 contributed to higher oxidative stability.

• High total unsaturation of 91.7% dominated by monounsaturated fatty acids and minimal saturated content, reducing crystallization at low temperatures resulted in showing the lowest cloud points and pour points for WCO B-2. Under unelectrified conditions, WCO B-3, B-4 exhibited 17% and 23% lower average coefficient of friction and achieved lower average wear depths than RSO.

• Under electrified conditions, all batches experienced increased wear and oxidation; however, WCO B-4 maintained the lowest average wear depth despite some frictional instability. SEM-EDS revealed more severe oxidation and reduced material transfer under electrified test samples compared to unelectrified ones. Oxidative and abrasive wear were dominant, with electrical stress accelerating surface degradation. Raman spectroscopy revealed a notable difference in oxide formation mechanisms under electrified and non-electrified conditions, which played a crucial role in altering tribological behavior under electrified conditions.

Overall, WCO demonstrated tribological performance comparable to or exceeding that of RSO, highlighting their potential as a sustainable base oil for electrified drivetrains with chemical modification. Future efforts are focused on additization and further chemical modification of WCOs to further enhance their oxidation, low-temperature flow, and tribological behavior and followed by developing fully formulated lubricants for future electric vehicles.

## Conflicts of interest

There are no conflicts to declare.

## Data Availability

The authors confirm that the data supporting the findings of this study are available within the article. Additional raw and/or derived data supporting the findings of this study can be available from the corresponding author [S.R.] on request.
